# Classification of Soccer and Basketball Players’ Jumping Performance Characteristics: A Logistic Regression Approach

**DOI:** 10.3390/sports7070163

**Published:** 2019-07-04

**Authors:** Christos Chalitsios, Thomas Nikodelis, Vassilios Panoutsakopoulos, Christos Chassanidis, Iraklis Kollias

**Affiliations:** Biomechanics Laboratory, Department of Physical Education and Sports Sciences, Aristotle University of Thessaloniki, 54124 Thessaloniki, Greece

**Keywords:** vertical ground reaction force, sport specificity, impulse, rate of force development, biomechanics

## Abstract

This study aimed to examine countermovement jump (CMJ) kinetic data using logistic regression, in order to distinguish sports-related mechanical profiles. Eighty-one professional basketball and soccer athletes participated, each performing three CMJs on a force platform. Inferential parametric and nonparametric statistics were performed to explore group differences. Binary logistic regression was used to model the response variable (soccer or not soccer). Statistical significance (*p* < 0.05) was reached for differences between groups in maximum braking rate of force development (RFD_Dmax_, U_79_ = 1035), mean braking rate of force development (RFD_Davg_, U_79_ = 1038), propulsive impulse (IMP_U_, t_79_ = 2.375), minimum value of vertical displacement for center of mass (S_BCMmin_, t_79_ = 3.135), and time difference (% of impulse time; Δ_Τ_) between the peak value of maximum force value (F_Umax_) and S_BCMmin_ (U_79_ = 1188). Logistic regression showed that RFD_Davg_, impulse during the downward phase (IMP_D_), IMP_U_, and Δ_Τ_ were all significant predictors. The model showed that soccer group membership could be strongly related to IMP_U_, with the odds ratio being 6.48 times higher from the basketball group, whereas RFD_Davg_, IMP_D_, and Δ_Τ_ were related to basketball group. The results imply that soccer players execute CMJ differently compared to basketball players, exhibiting increased countermovement depth and impulse generation during the propulsive phase.

## 1. Introduction

The main focus of athletic training at the competitive level is to improve the athlete’s specific and relevant abilities that are essential to the sport. Players in sports such as basketball and soccer usually perform repetitive tasks such as jumps, rapid changes of direction, and intense accelerations or decelerations [1,2]. The execution of these movements is primarily based on the capacity of the musculoskeletal system to produce power and impulse [3]. Vertical jumping is a fundamental skill that may distinguish top performers in both sports and, for that reason, it represents a training goal for strength and conditioning coaches. Various training models are usually adopted by professionals in order to increase that capacity [4,5]. The unloaded countermovement jump (CMJ) without arm swing is one of the most popular tests to assess lower limb performance [6]. Studies have shown that the observed increase in jump height after training represents a valid and positively contributing factor to the improvement of sporting performance [7,8]. Yet, assessing athletic performance only in terms of jump height is somewhat simplistic as it does not offer an inside view of the mechanisms defining performance.

Vertical jumping force–time characteristics are quite different between soccer and basketball [9]. The main difference is the frequency and timing in which athletes are called upon to perform jumps. In soccer, the playing fields are larger and there is no time restriction for specific game actions to occur, thus, there is more time for prediction and reaction, while for basketball, the opposite is true. These differences may affect the way that players regulate their jumping strategy and trigger the adoption of different training protocols.

Indeed, training background has an effect on the pattern of vertical force production [10]. The use of principal component analysis to explore force–time series during vertical jumping showed that the extracted components could discriminate different sporting backgrounds. More specifically, it revealed that sport-specific kinetic profiles exist and they are based on the utilization of the force and temporal parameters in a sport background combination [9,11,12,13]. Although principal component analysis has been used extensively for exploring the underlying structure of observed patterns between different groups of interest, this method aims to analyze each variable under the assumption that features with high variance are more likely to achieve a good split between the classes. This technique presents a geographical representation of the trend towards a principal component in the n-dimensional space. Nevertheless, it does not provide a numerical expression for the possibility of an individual to belong in a sports group according to the features that comprise his/her performance. 

The adoption of a simple, yet powerful-enough classification model, which considers every independent variable impact on the response variable, may provide alternative insights into the differences among examined populations and, more importantly, provide interpretable and usable results. One such method is logistic regression. This statistical technique is often used for the probability estimation of the dependent variable from one or more predictor variables, and presents a useful and efficient tool to assess independent variable contributions to a binary outcome. Classifying the overall mechanical profile of a jump execution in such a way, regardless of the statistical differences that may exist in individual parameters, may prove useful for training, recruiting, and even injury-preventing purposes. In fact, there are already such examples in gait biomechanics [14]. 

Furthermore, up until now, such classification analysis of jumping mechanical profiles has not been conducted, at least to the extent of our knowledge and after intensive research. Instead, the comparative literature is limited to descriptive differences on selected variables of CMJ. For example, soccer and basketball players do not differ significantly in terms of jump height [15]. Such results do not offer useful information to strength coaches about the underlying kinetic mechanisms of jumping strategy that may be imposed by the sport specificity. The purpose of the present study was to combine CMJ kinetic data with the statistical classification tool of logistic regression in order to test the hypothesis that different mechanical profiles are sports-dependent.

## 2. Materials and Methods

### 2.1. Study Design

A cross-sectional design was adopted to explore various features of vertical ground reaction force (GRF_V_)–time curve during CMJ. Statistical inference and classification modeling were used to investigate the differences that could be identified due to the sporting background. Various biomechanical factors on which jumping performance is based were considered in both sports groups. 

### 2.2. Participants

In this study, 81 male adult athletes performing in top-level professional leagues in Greece for basketball (n = 39, age 28.9 ± 3.5 years, body mass 97.4 ± 10.6 kg, height 1.97 ± 0.08 m) and soccer (n = 42, age 26.4 ± 4.3 years, body mass 77.0 ± 9.3 kg, height 1.82 ± 0.07 m) were tested. Participants were inspected for any muscular dysfunction that may have occurred during the three months before testing. In any case, athletes with typical injuries caused by impacts during practice or competition had completely recovered at the time of the measurements. Testing took place during the last week of the pre-season, where the training volume was intentionally lower to minimize accumulating fatigue. Participants abstained from team practice one day before testing. Prior to their participation, all individuals signed informed consent forms regarding the risks and benefits of the investigation according to the Institutional Ethics Committee Guidelines.

### 2.3. CMJ Testing

All participants performed a series of three CMJs on a triangular dual force plate system (k-Delta, K-Invent Biomecanique, Orsay, France) that incorporated three 1D force sensors on each plate. Before testing, participants carried out a 10 min standardized warm-up that consisted of slow pace running on a treadmill for 6 min at a constant velocity of 2 m·s^−1^ without load or inclination and combined with static and dynamic stretching. After a thorough explanation and physical demonstration, each individual performed the test based on standardized instructions “to jump as high and as fast as possible”. Three submaximal jumps on the force plates followed the warm-up, for checking technical execution. All jumps were executed with hands at the akimbo position until landing and stabilization. Each participant performed a total of three jumps without any restriction for countermovement depth. A time interval of 1.5 min was set between trials to avoid any fatigue. The trial with the highest value for jump height was further analyzed. All testing was performed between 16:00 and 18:00 pm.

### 2.4. Data Recording and Analysis

GRF_V_ acquired from the force plate was sampled at 500 Hz and the force plate’s accompanying software was used to obtain the raw data values. GRF_V_ from each platform was summed to create the total force–time signal. Raw data were filtered using a 2nd order Butterworth filter and the cutoff frequency was set at 20 Hz. Force data were used to compute the variables for braking and propulsive phases. The braking phase was defined from the initiation of the movement (force below 95% of body weight) until the point where vertical displacement reached its minimum value. The propulsive phase started immediately after the end of the braking phase until take-off. Jump height calculation was derived using the impulse-momentum theorem [16]. Recorded data were used to calculate kinetic and kinematic variables such as maximum braking rate of force development (RFD_Dmax_), mean braking rate of force development (RFD_Davg_), braking impulse (IMP_D_), propulsive impulse (IMP_U_), maximum force value (F_Umax_), mean value of force (F_Uavg_) over the propulsive phase, peak power (P_Umax_), mean power (P_Uavg_), the minimum value of vertical displacement (S_BCMmin_), and the time difference (% of impulse time) between the peak value of F_Umax_ and S_BCMmin_ (Δ_T_). All variables were expressed as units per body mass, except for RFD_Dmax_ and RFD_Davg_. The value of S_BCMmin_ was scaled to body height. All analyses were stored using MATLAB (2015b, Mathworks, Natick, MA, USA) and Signal Processing Toolbox.

### 2.5. Statistical Analysis

Statistical procedures were performed with R v3.2.2 (R Foundation for Statistical Computing, Vienna, Austria). The average value of the standard error of measurement (SEM) between the three trials, concerning jump height, was 2.5 ± 1.1 cm, which accounts for ~2% of maximum hump height. Distribution properties of the data were checked using the Shapiro–Wilk test. If distributions between groups did not reject the null hypothesis of the normality test, an independent samples Student’s *t*-test was applied over all variables to check for differences in the means between the two groups. If data were not normally distributed, a Mann–Whitney *U* test was carried out. Cohen’s effect sizes *d* and *r* were calculated and reported for parametric and nonparametric tests respectively.

Multivariable logistic regression analysis was performed to classify the binary outcome of group membership (Basketball = 0, Soccer = 1). Classification threshold was set to 0.5. The logistic regression model was estimated using a logit link function, assuming a binomial distribution for the outcomes. Model development was based on a backward elimination strategy using the Akaike information criterion (AIC) as a selection metric. The model with the lowest AIC was selected for further analysis. Variance inflation was used for multicollinearity checking and variables that inflated above 5 were removed. Goodness-of-fit was obtained based on the Stukel test [17,18]. The test is based on the null hypothesis that there are no deviations from the logit link function. The logistic equation was solved for each participant to determine into which group he would be classified. Discrimination ability was quantified based on the area under the curve (AUC) which measures the area under the receiver operating characteristic (ROC) curve. An AUC value of 1 (100%) corresponds to perfect discrimination and 0.5 (50%) to random chance. The application of a resampling technique is recommended for the analysis of small sample sizes [19]. Thus, for the internal validation of the model, a bootstrap approach was carried out to estimate the performance of the classifier and to avoid overfitting [20]. For all statistical tests, an alpha level of 0.05 was set.

## 3. Results

**Figure 1:**
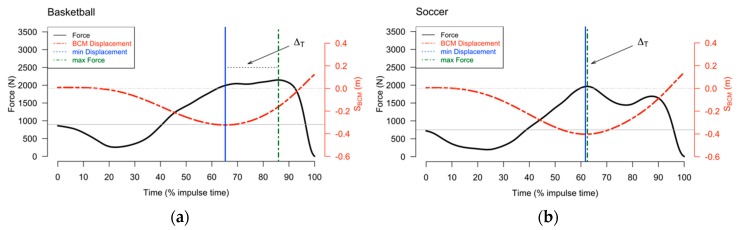
Example of characteristic difference in Δ_Τ_: (**a**) typical force–time curve of a basketball player; (**b**) typical force–time curve of a soccer player. Vertical lines represent the maximum value of countermovement depth (solid blue line) and maximum value of force (green dashed line). The grey horizontal line indicates subjects’ body weight, and the dashed grey horizontal line indicates the zero value for S_BCM_.

Results revealed statistically significant differences in several of the examined variables. Descriptive statistics are shown in Table 1. Statistical significance was reached in RFD_Dmax_ (U_79_ = 1035, r = 0.88) and RFD_Davg_ (U_79_ = 1038, r = 0.87), with soccer players exhibiting lower values compared to basketball players. Furthermore, significantly greater values were observed in IMP_U_ (t_79_ = −2.375, d = 0.54) and S_BCMmin_ (t_79_ = 3.135, d = 0.7), but not for Δ_Τ_ (U_79_ = 1188, r = 0.69) in the soccer group, as shown in Figure 1.

After the variable elimination process, the final logistic regression model was built using only RFD_Davg_, IMP_D_, IMP_U_, and Δ_Τ_ (Table 2) where all predictors contributed significantly (*p* < 0.05). Stukel’s test for goodness-of-fit was not significant (*p* = 0.627).

**Figure 2:**
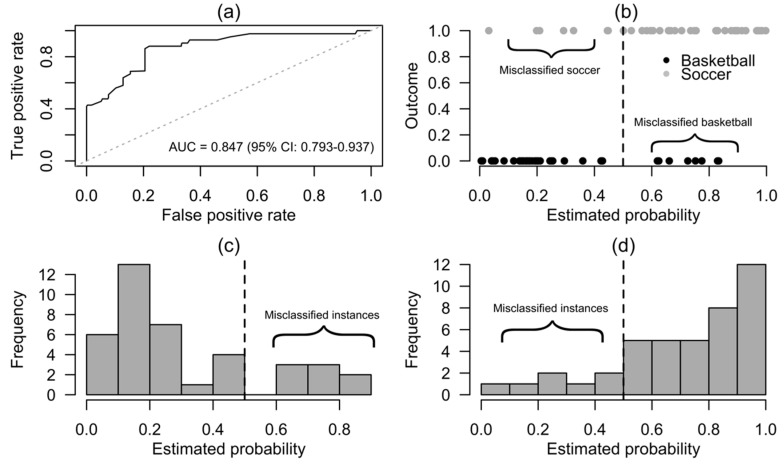
Diagnostic plots of the logistic regression model predicting group membership: (**a**) Model’s AUC performance; (**b**) Scatterplot of the respective classes probability; (**c**) Histogram—basketball probability (cutoff threshold, dashed line); (**d**) Histogram—soccer probability (cutoff threshold, dashed line).

The proposed model was able to discriminate the groups (Figure 2) with an in-sample AUC of 0.87. After bootstrap, the AUC value was 0.847. 

Exponentiation of the log of odds to obtain the odds ratio for the predictor variables revealed that the odds ratio for soccer group membership increased by 6.48 times for every N·s increase in IMP_U_ during CMJ. On the contrary, for every 1kN·s^−1^ increase in RFD_Davg_, the odds ratio of any observation to be classified as soccer player decreased multiplicatively by 0.3 times. Similarly, for unit increases and regarding the variables IMP_D_ and Δ_Τ_, the odds ratio of soccer group membership decreased multiplicatively by 0.07 and 0.85 times, respectively. All four variables of the regression reached a Nagelkerke pseudo R^2^ value of 0.506, suggesting that 50.6% of the total variance was explained by RFD_Davg_, IMP_D_, IMP_U_, and Δ_Τ_.

## 4. Discussion

In this study, we aimed to establish a classification method to confirm the assumption that features of GRF_V_ during CMJ can discriminate athletes according to their sporting background. The logistic regression approach was preferred because it provides interpretable results and, at the same time, it performs well enough in terms of predictive ability. The experimental results appear supportive of the feasibility of the proposed method. Descriptive statistics showed that basketball players appeared to produce significantly higher values for RFD_Davg_ and Δ_Τ_, while soccer players showed significantly higher values for IMP_U_ and S_BCMmin_. These significant differences will be discussed in relation to the logistic regression. The final model of logistic regression was built using only four variables out of the full model. The selected variables were RFD_Davg_, IMP_D_, IMP_U_, and Δ_Τ_. Stukel’s goodness-of-fit failed to reject the null hypothesis and showed no evidence of poor fit [21]. 

The bootstrapped AUC (0.847) value for the final model denotes that this relatively simple, four-variable final model displayed very good predictive power for the distinction of the original groups. The final model demonstrated that membership to the soccer group could be strongly related to IMP_U_, with the odds ratio being 6.48 times higher from the basketball group. This implies that soccer players used a different kinetic pattern during the execution of CMJ, based on generating high impulses during the propulsive phase of the movement. This indication is in accordance with other studies where participants that exhibited greater countermovement depth also achieved greater values in IMP_U_ [22,23] and, consequently, greater jump height values compared to others. This is also supported by the paired comparisons for the selected variable. 

The model also displayed that RFD_Davg_, IMP_D_, and Δ_Τ_ were significant predictors of group classification. The result that RFD_Davg_ is associated with basketball players is indicative of their muscle–tendon system’s capacity to develop force quickly during the stretching phase of the stretch–shortening cycle [12] using a decreased countermovement depth. Nevertheless, the aforementioned reference did not consider the unloading phase to be part of the downwards impulse phase, thus introducing a bias to their calculations [16]. Ugrinowitch [10] also proposed that athletes, in sports where time constraints are present, try to apply force with large acceleration values in order to maximize jumping height.

Another interesting finding is that the increasing trend of Δ_Τ_ value was related to the basketball group. The time used for the execution of the jump has been shown to affect the mechanical properties of performance even through verbal instructions [24]. Moreover, it seems that for basketball players, the moment of reaching minimum displacement (S_BCMmin_) is more distal from the time point of F_Umax_ application compared to soccer players. The jumps that basketball players usually perform during practice or in competition are not maximal as they are constrained to act before opponents. For example, consecutive rebounding, block and rebound, and shooting to beat the buzzer are totally different from a timing perspective compared to jumps in soccer, and especially regarding the time coupling interaction with the opponent. 

This leads to jumps that are executed quickly, without large countermovement action, and with high dependence on force production from the ankle plantar flexors [25,26]. Indeed, Salles et al. [22] proposed that the appearance of F_Umax_ close to the end of the propulsive phase during CMJ is related to increased calf muscle activation. At that specific point in time, hip and knee joints are already extended due to decreased countermovement and short duration of push-off, preventing their extensors reaching maximal activation and from thus producing maximum force and impulse [27,28]. The joint that can continue the contribution to the force production is the ankle, as it is not yet fully plantar-flexed [22]. 

Soccer players, on the other hand, seem to utilize this specific parameter (Δ_Τ_) in the opposite way. It seems that for soccer players, the time points of F_Umax_ and S_BCMmin_ are occurring almost at the same time, and they consistently performed CMJs with greater depth in comparison to basketball players. Bobbert et al. [28] showed that the lower the position of the center of mass was before the upward push-off phase, the later the activation of plantar flexors occurred. This was indicative of greater activation for knee and hip flexors. Maximal jumping in soccer is a crucial factor for claiming the ball. Soccer players have more time at their disposal for performing a vertical jump due to the larger distances they are called to act upon in a soccer field. The aforementioned features may be the sport specific regulators of that variable (Δ_Τ_). 

Furthermore, soccer players exhibit larger push-off durations and IMP_U_ in order to maximize their jump height. Fukashiro and Komi [25] stated that this kinetic pattern is associated mainly with energy contribution from the hip/knee joint bi-articular muscles and minimal activity from muscles in the ankle. Similar findings from Vanrenterghem et al. [29] demonstrate that the ankle joint contributes only 23% of the necessary energy generation of a maximal jump. A connection between increasing the total work in order to increase jump height and the increased contribution from the large proximal muscles has also been reported in the literature [30]. Recently, this statement has been associated with increases in hip and knee peak angles, indicating transitions from an ankle-centered strategy to a hip/knee movement strategy [26]. 

The confidence interval for the odds ratio (1.02–41.1) of IMP_U_ is wide. Such wide confidence intervals often occur due to small sample sizes, explanatory variables with a narrow distribution, or data sparsity. Sparse data are often present in research settings, especially in binary logistic regression which has been identified as a condition that needs to be taken under scrutiny when the original dataset lacks sufficient case numbers for some combinations of explanatory and outcome variables [31]. The sample of the present study was rather homogeneous with athletes of the same level and, therefore, it is unlikely that inflation in the confidence interval was generated from sparse data. In any case, an increased sample size would narrow down the confidence interval for the odds ratio estimation of IMP_U_.

CMJ testing is a widespread procedure for assessing performance in both soccer and basketball, however, the specific movements and training regimes are usually different between these sports. The results of the present study provide useful information on how CMJ mechanisms may differ between athletes of different sports, and which kinetic variables indicate stronger relationships with each sport. The information may be used for training and testing purposes, in case an athlete displays a force–time curve with less sport-specific features. For example, a soccer athlete with a higher braking RFD_Davg_ than IMP_U_ may be advised to increase push-off durations until take-off, or adjust training contents to generate more power through knee and hip flexors.

## 5. Conclusions

Overall, the challenge of specifying the kind of analysis that is optimal for assessing jumping performance calls for further research. The main advantage of logistic regression is that it usually can avoid any confounding effects by analyzing the associations of all variables of interest together [32]. The aforementioned statistical framework clearly indicates differences in vertical force application patterns between soccer and basketball players. This finding is a hint for further investigation regarding, for example, the assumption that jumping performance may be related to “position–role” effects during competition and practice.

## Figures and Tables

**Table 1:** Descriptive statistics for the examined variables also used in the baseline model (mean ± SD, CI 95% for the difference in means, *p* value).

Variable	Basketball (n = 39)	Soccer (n = 42)	Difference	CI 95%		*p*
	Mean ± SD	Mean ± SD		Lower	Upper	
RFD_Dmax_ (kN·s^−1^)	**13.87 ± 5.11^a^**	11.91 ± 5.31	1.96	0.08	4.02	**0.041**
RFD_Davg_ (kN·s^−1^)	**5.67 ± 2.03^a^**	4.67 ± 1.65	1.00	0.04	1.67	**0.038**
IMP_D_ (N·s)	3.93 ± 0.54	4.04 ± 0.81	−0.11	−0.27	0.22	0.921
IMP_U_ (N·s)	5.25 ± 0.47	**5.49 ± 0.45^b^**	−0.24	−0.45	−0.04	**0.020**
F_Umax_ (N·kg^−1^)	24.47 ± 2.71	24.12 ± 1.94	0.35	−0.94	1.10	0.991
F_Uavg_ (N·kg^−1^)	19.97 ± 1.48	19.89 ± 1.59	0.08	−0.61	0.70	0.868
P_Umax_ (W·kg^−1^)	53.94 ± 6.18	55.10 ± 6.31	1.16	−4.18	1.26	0.348
P_Uavg_ (W·kg^−1^)	29.96 ± 3.79	30.81 ± 3.91	−0.85	−2.60	0.78	0.344
S_BCMmin_ (m)	−0.16 ± 0.04	**−0.19 ± 0.04^b^**	0.03	0.01	0.04	**0.002**
Δ_Τ_ (%)	**13.56 ± 9.3^a^**	6.44 ± 8.3	7.12	1.78	14.13	**0.000**

a: Mann–Whitney *U* test, b: Independent *t*-test.

**Table 2:** The output of the logistic regression. Log odds, odds ratio, and confidence intervals (95% CI) for odds ratio and *p* values of the coefficients.

Variable	Log Odds	SE	Odds Ratio	CI 95%	*p*
RFD_Davg_	−1.194	0.33	0.3	(0.16–0.58)	0.000
IMP_D_	−2.639	0.85	0.07	(0.01–0.37)	0.002
IMP_U_	1.869	0.94	6.48	(1.02–41.1)	0.047
Δ_Τ_	−0.161	0.03	0.85	(0.79–0.92)	0.000

Note: Nagelkerke’s R^2^ = 0.506.
